# Computational Modeling and Analysis to Predict Intracellular Parasite Epitope Characteristics Using Random Forest Technique

**Published:** 2020-01

**Authors:** Amir JAVADI, Ali KHAMESIPOUR, Farshid MONAJEMI, Marjan GHAZISAEEDI

**Affiliations:** 1 Department of Health Information Management, School of Allied Medical Sciences, Tehran University of Medical Sciences, Tehran, Iran; 2 Department of Medical Social Sciences, Faculty of Medicine, Qazvin University of Medical Sciences, Qazvin, Iran; 3 Center for Research and Training in Skin Diseases and Leprosy, Tehran University of Medical Sciences, Tehran, Iran; 4 Ministry of Health and Medical Education, Tehran, Iran

**Keywords:** Computational model, Immunogenic peptides, Intracellular parasites

## Abstract

**Background:**

In a new approach, computational methods are used to design and evaluate the vaccine. The aim of the current study was to develop a computational tool to predict epitope candidate vaccines to be tested in experimental models.

**Methods:**

This study was conducted in the School of Allied Medical Sciences, and Center for Research and Training in Skin Diseases and Leprosy, Tehran University of Medical Sciences, Tehran, Iran in 2018. The random forest which is a classifier method was used to design computer-based tool to predict immunogenic peptides. Data was used to check the collected information from the IEDB, UniProt, and AAindex database. Overall, 1,264 collected data were used and divided into three parts; 70% of the data was used to train, 15% to validate and 15% to test the model. Five-fold cross-validation was used to find optimal hyper parameters of the model. Common performance metrics were used to evaluate the developed model.

**Results:**

Twenty seven features were identified as more important using RF predictor model and were used to predict the class of peptides. The RF model improves the performance of predictor model in comparison with the other predictor models (AUC±SE: 0.925±0.029). Using the developed RF model helps to identify the most likely epitopes for further experimental studies.

**Conclusion:**

The current developed random forest model is able to more accurately predict the immunogenic peptides of intracellular parasites.

## Introduction

Historically, the most effective public-health prevention against infectious disease is vaccination. Development of an effective vaccine against any disease is a major breakthrough to control the disease. There have been tremendous efforts to develop vaccines against infectious and non-infectious diseases, but yet no vaccine is available against many infectious diseases. The development of a new vaccine from theory to practice is a complex process task. Preclinical studies to develop a vaccine are a long process, time-consuming, needs enough funds and infrastructure, which are not available in the regions of the world, which suffer from the infectious diseases the most. Emerging modern technology and computational models in biomedicine have provided new horizons for discovering, and designing vaccines. Using *in-silico* approach, the designed epitopes might be used and tested experimentally in the preclinical setting. Nowadays, using *in-silico* approach has been advanced rapidly and assists in different aspects of biomedical sciences. In *in-silico* approach, vaccine logically is designed using computational algorithms and evaluate using computer simulation (1–3). Using *in-silico* approach is a shortcut method to identify novel immunogenic peptides for the development of a vaccine prior to in vitro and in vivo evaluation.

Several computational methods, including binding motifs (BM), quantitative matrices (QM), machine learning algorithms (ML), evolutionary algorithms, linear programming and hybrid methods are usually used to predict the class of peptides (4–6). The computational methods mostly distinguish the peptides based on amino acid properties. Among the computational methods, ML is more commonly used to identify the class of peptides and design epitope-based vaccines for the prevention and/or possibly treatment of infectious and non-infectious diseases (7). Some of the common supervised ML algorithms for pattern recognition include support vector machine (SVM), neural networks (NN), naïve Bayes, decision tree (DT), random forest (RF), and hybrid methods (8,9). Among the above-mentioned methods, RF is the more popular ML approach, due to the fact that it is easy to understand, handy to use, interpretation and robustness. In RF algorithm, various decision trees with a high diversity between the individual trees were generated in the forest. Every one of the created decision trees independently predicts the class of the peptides. The diversities of the trees are controlled using bootstrap replacement sampling from the training dataset and a subset of the features is randomly selected. Then, the final decision is made based on the majority of the votes of the aggregated predicted trees (10–15).

The aim of the current study was to develop computational tools based on ensemble random forest machine learning model to facilitate Th1 epitopes identification to be used as the vaccine candidate for intracellular parasites.

## Materials and Methods

The methods used in the current study for the data collection, peptide properties extraction, data processing, and the development of RF model are as follows:

### Data resources

The sequences of the proteins were retrieved from UniProtKB/Swiss-Prot database http://www.uniprot.org/ (16,17). T cell epitopes were retrieved from Immune Epitope Database (IEDB) http://www.iedb.org/ (18). Access to both databases are free. The date of the data retrieval is Apr 18, 2017.

### Data preparation

From 6,223 MHC class II T cell epitopes retrieved from IEDB database, 3,200 epitopes with a length of *9- to 21-mer* were selected from 524 antigens previously showed to be immunogenic and as such were marked as positive assays epitopes. Gibbs sampler method (19,20) was used to align *9-mer* core-binding motif and stored as epitope dataset class. To select non-epitope peptides, the proteins which contain epitopes with define sequences were retrieved from UniProtKB/Swiss-Prot database, after removing the epitopes, the remaining sequences were scanned using windows size of *9-mer* to extract non-epitope peptides. The non-redundant extracted peptides were stored as the non-epitope dataset class. Two stored datasets were used to train, validate and test the RF model.

### Peptide descriptor extraction

The properties used to develop the model are peptide AA composition (AAC) and AA physico-chemical properties (AAPP). The AAC for each peptide was calculated with the following equation where *k* is one of each 20 AA:

$$\mathrm{AAC}(k)=\frac{\mathrm{Frequency}\;\mathrm{of}\;\mathrm{AA}(k)}{\mathrm{Length}\;\mathrm{of}\;\mathrm{peptides}\;=1,...,20\mathrm{AA}\;\mathrm{index}},k$$

The AAPP used to identify the class of peptide are as follows:

The distribution of residue AA in each position, aliphatic index (21), hydropathy scale (22), polarity scale (23), isoelectric point (PI) (24), net charge, number of bulky AA (Leu, Ile, Phe, Try, Tyr, Val), number of less bulky AA (Ala, Arg, Asp, Asn, Cys, Glu, Gln, Gly, His, Lys, Met, Pro, Ser, Thr) (25), chemical characteristic of the peptides (aromatic, aliphatic, sulfur, hydroxyl, and amide), and the number of potential side-chain hydrogen bonds (donor, acceptor, both, and Non) (26).

### Model development

The MATLAB ver. 2014 software was used to develop the RF model, RF is an ensemble-learning approach usually used for classification and regression. RF combines various classifications DT is used to produce a more accurate classification. Bootstrap aggregation algorithm was used to create the ensemble DT classifiers. Each classifier independently predicts the class of peptides and the majority vote on the DT classifiers defines class of peptides in RF model. In this study, Gini’s Diversity Index (GDI) was used to measure the node impurity, and feature with the highest GDI was selected as the split feature in the node. The performance of RF algorithm depends on the tuning of a number of hyper parameters. The optimal hyper parameters were distinguished using assign multiple values to develop a suitable model. The 5-fold cross-validation was used to evaluate and tune the hyper parameters. The values assigned to each parameter are as follows: The maximum number of random ensemble trees (*n-Tree*) in RF model is set to 2,000. The number of predictors used to split the appropriate node (*m-try*) was set to 9 (square root of features number in the dataset). The minimum size of the leaf node (*node-size*) was set to 2. The maximum growth depth (*tree-Depth*) for each RT was set to 100.

### Performance evaluation

The collected data set was randomly divided into three parts; 70% of the data was used to train; 15% of the data was used to validate, and the rest 15% of the data was used to test the model. The performance of the model was calculated by accuracy, sensitivity, specificity, positive predictive value (PPV), negative predictive value (NPV), error rate, and area under the ROC (AUC) (27–29).

### Statistical analysis

The Cohen's kappa statistics was used to quantify degree of agreement and assess reliability of the model. The Pearson’s chi-square, McNemar’s, Wald, and Z test were used to analysis of data (30,31). The probability values less than 0.05 were considered statistically significant. The statistical analyses were performed using SPSS 16.0 (SPSS Inc., Chicago, IL, USA).

## Results

From the 3,200 epitopes, 1,264 non-redundant *9-mer* core-binding motif and 1,264 similar nonepitope peptides were used to train, validate, and test the model.

**Fig. 1:**
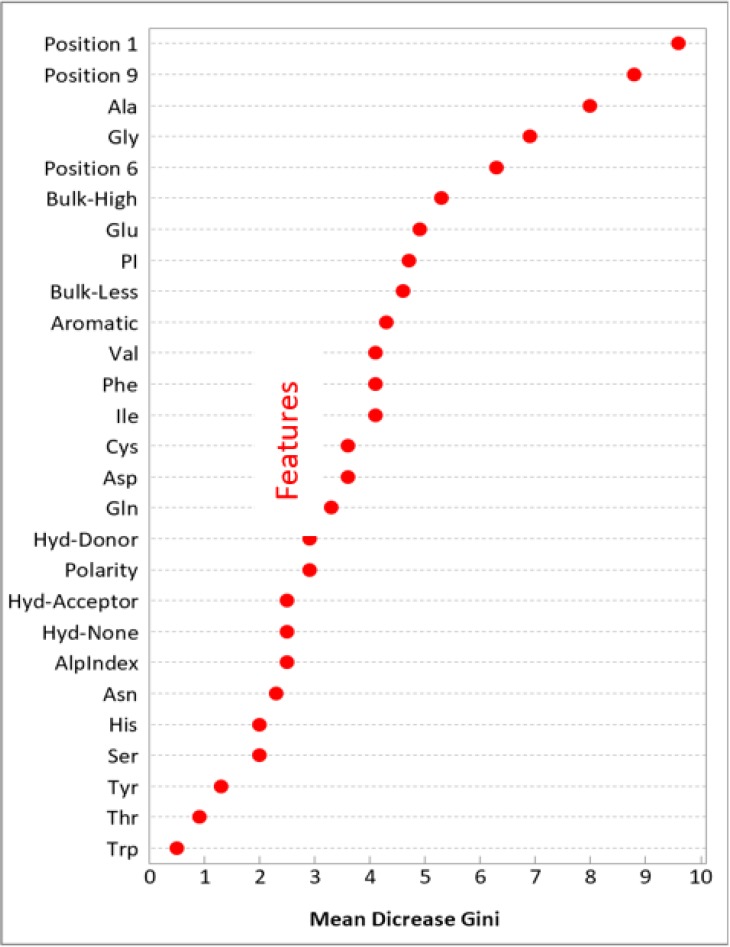
Features importance plot

The feature selection is an important step to develop RF model. In this step, the impurity features were included and the noisy and redundant features were excluded to improve the performance. Figure 1 shows the feature importance score distribution with the positive score of the peptide properties. From 59 features, twenty-seven features are positive score and fourteen are detected as having the greatest effect to discriminate the class of peptides (criteria greater than 40% was considered as cut off). The AA residue at position 1 is the highest rank feature to identify the class of peptide with a 99% score. The next feature is AA residue at position 9 with 91% importance score. The number of alanine and glycine are 82%, and 70% importance, respectively. The AA residue at position 6 is 64% importance score. The number of bulky AA and glutamic acid in the peptide is 53%, and 48% importance, respectively. The PI index is a 46% importance score. The number of bulky less and aromatic AA in the peptide is 45%, and 42% importance, respectively. The numbers of isoleucine, phenylalanine, and valine AA in the peptide was 40% importance. The other features in the model are less important, with percentages of 34% to 5% (Fig. 1).

The accuracy of the RF model to train, validate, and test the dataset are 96.7%, 95.8%, and 91.6%, respectively. Therefore, only at maximum 8.4% of the data was incorrectly classified.

The minimum sensitivity and specificity for the 3 datasets are 92.6% and 90.5%, respectively, which means the RF model correctly detects at least 92.6% of the epitopes and 90.5% of non-epitopes. The minimum PPV and NPV for each of the 3 datasets are 90.7% and 92.5%, respectively, that means the RF model categorized the epitopes correctly at least in 90.7% of the epitopes in this class and categorized correctly at least 92.5% of nonepitope class in this class (Table 1).

**Table 1:** Measures of performance RF model for each data set

***Partition***	***Train (n=884)***	***Validation (n=190)***	***Test (n=190)***
Accuracy	855 (96.72%)	182 (95.79)	175 (92.51)
Error Rate	29 (3.28%)	8 (4.21%)	15 (7.49%)
Sensitivity	97.51	97.89	92.63
Specificity	95.93	93.68	91.58
PPV	95.99	93.94	91.67
NPV	97.47	97.80	92.55
Kappa coefficient	0.934±0.012	0.916±0.029	0.842±0.038

**Fig. 2:**
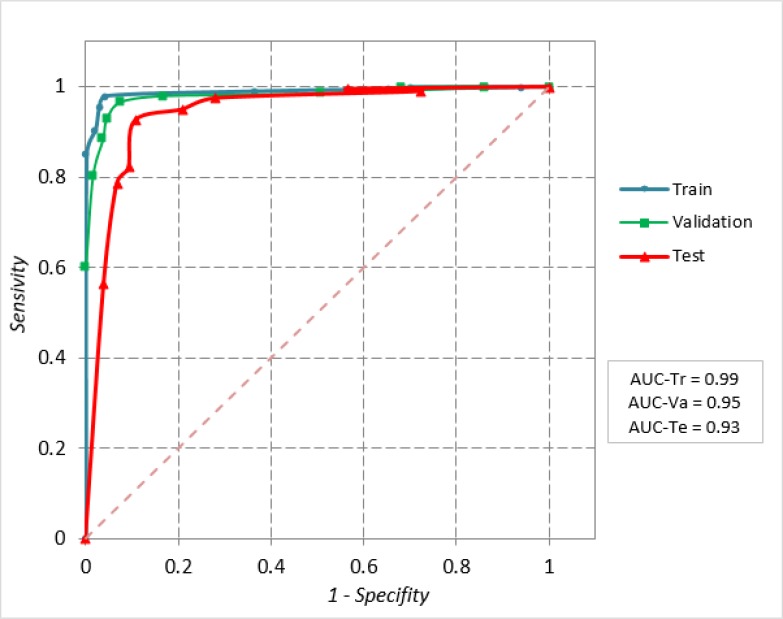
ROC curve and AUC for RF model

The area under the ROC curve that shows the expected performance of the RF model for the train dataset is 0.995±0.002 (95% CI: 0.99 to 1.0), validate dataset is 0.958±0.021 (95% CI: 0.92 to 1.0), and the test dataset is 0.925±0.029 (95% CI: 0.87 to 0.98) (Fig. 2). The AUC values show that the RF model is able to discriminate the class of peptide in the three datasets (*P*<0.001). The value of Cohen's Kappa for the test dataset is 0.842 (95% CI: 0.78 to 0.93), which means the results of RF developed model in 70.9% are reliable (*P*<0.001).

Table 2 shows the description of the decision rules (DRs) extracted to classify the peptides, ordered by the rule accuracy. The developed RF model obtained 6 rules with accuracy from 88% to 97%. The DRs 1, 4, 5, and 6 identified the non-epitope class with rule accuracy of 97%, 93%, 90%, and 88% respectively. The DRs 2 and 3 identified the epitope class with rule accuracy of 97% and 95%, respectively. The number of features in rules varies from 10 to 17.

**Table 2:** Top Decision Rules for identify class of peptide

***No***	***Decision Rule***	***Class***	***Rule Accuracy***
1	(P1 = {D,E,H,K,N,R,S,T}) and (P9 = {D,E,G,H,K,N,Q,R,S,T,V})	Non Epitope	0.978
2	(P1={A,F,I,L,M,V,W}) and(P6={A,C,H,R,V}) and(P9={A,C,E,F,I,K,L,M,Q,R,S,V,W}) and(PI >= 3.7 and PI<=6.5)	Epitope	0.970
3	(P1 = {A,C,F,I,L,M,S,V,W}) and (P9 = {A,C,D,F,I,L,Q,S,V} and (Aromatic >= 1.0)	Epitope	0.949
4	(P1 = {D,E,G,H,K,Q,R,S,T}) and (P9 = {A,C,D,E,G,H,K,M,N,Q,R,S,T,V}) and (Bulk-Less > 6.0) and (A > 1.0))	Non Epitope	0.929
5	(P1 = {D,E,G,H,K,N,Q,R,S,T}) and (P9 = {A,D,E,F,G,H,K,N,Q,R,S,T,V}) and (Bulk-Less > 5.0)	Non Epitope	0.900
6	(P1 = {D,E,G,H,K,N,Q,R,S,T}) and (P9 = {D,E,G, K,N,Q,R,S,T})	Non Epitope	0.879

Among the 17 features, the AA at position 1 and 9 are the most predictive capacity in all the rules. The most frequent AA at position 1 in nonepitope peptides is (D, E, G, H, K, N, Q, R, S, and T) and at position 9 is (A, C, D, E, G, H, K, M, N, Q, R, S, T, and V). The alanine at position 1 and glycine at position 9 in non-epitope class are non-polar and others are polar AA residues. The most frequent AA at position 1 in epitope class is (A, C, F, I, L, M, S, V, and W) and at position 9 is (A, C, D, E, F, G, H, I, K, M, Q, R, and V). The glycine at position 1 is neutral and others AA are hydrophobic. The AA at position 9 is either of 3 hydropathy class (hydrophobic, neutral, or hydrophilic) (Table 2).

Along with AA type in positions 1 and 9 at DR4, the number of bulky less AA greater than 6 and the number of alanine AA greater than 1 is indicative of a non-epitope. Along with AA type in position 1 and position 9 at DR5, the number of bulky less AA greater than 5 is indicative that the peptide is a non-epitope. Along with AA type in position 1 and 9 at DR2, the AA type at position 6 including (A, C, H, R, V) and PI of peptide between 3.7 to 6.5 is an indicative that the peptide belongs to non-epitope class. Along with AA type in position 1 and 9 at DR3, the number of aromatic AA greater than 0 is an indication that the peptide belongs to epitope class.

## Discussion

The *in-silico* approach is a proper strategy to develop a novel epitope-based vaccine. In epitope-based vaccine design, identification of immunogenic peptide is the first and critical step. Using, computational approaches in vaccinology assist the researcher to predict the most likely immunogenic peptides for further complementary experimental studies which reduce the cost and the time to develop an effective vaccine. Many computational tools such as EpiTOP, MHCPred, ProPred, TEPITOPE, MHC2Pred, SVRMHC, SVMHC, RANKPEP, NetMHCII, and NetMHCIIpan have been developed to predict immunogenic peptides in a given protein. The performance of the developed tools varies and dependent on the type of algorithms and dataset used.

The EpiTOP and MHCPred tools use quantitative structure-activity relationship method (QSAR) to detect mathematical meaningful relationships between the peptide physicochemical properties, molecular structure and biological activities. The average of AUC for the EpiTop is 0.79 with a range of 0.72 to 0.89. MHCPred using a partial least squares multivariate statistical method to predict binder peptides to MHC molecules with overall accuracy of 0.79 (32,33). ProPred, uses quantitative affinity matrix (QAM) method to identify protein-protein interactions (PPIs), The average of AUC for ProPred is 0.76 with a range of 0.66 to 0.89 (34). TEPITOPE uses position-specific scoring matrix algorithms (PSSM) to score the conserved regions of the proteins. The average of AUC for TEPITOPE is 0.73 with a range of 0.67 to 0.77 (35).

MHC2Pred, SVRMHC, and SVMHC are SVM-base methods with different kernel functions (linear, polynomial and RBF) to predict the class of the peptides. MHC2Pred uses matrix optimization technique (MOT) to detect *9-mer* core-binding motif and predict promiscuous MHC class II binding core with overall accuracy of about 0.79 (33,36). SVRMHC uses quantitative SVM regression method to predict peptide-MHC binding affinities with an average of AUC=0.786 and a range of 0.74 to 0.83 (37). SVMHC predicts MHC-binding peptides with an average of AUC=0.76 and a range of 0.66 to 0.86 (38,39).

RANKPEP uses PSSM algorithms to score the conserved regions of the protein for both MHC class I and II molecules with an average of AUC=0.78 and a range of 0.54 to 0.93 (40). NetMHCII and NetMHCIIpan are network-based (NN) ensemble methods. These methods estimate the optimal peptide binding-core motif and neuron weighted connection. NetMHCII uses a set of individual networks for each MHC class, and NetMHCIIpan uses a single public NN model to predict epitope. The average of AUC for NetMHCII is 0.79, and a range of 0.71 to 0.85, and NetMHCIIpan is 0.858 and a range of 0.75 to 0.96 (41,42).

The range of AUC tools mentioned above is (0.73–0.86). The performance of RF developed model is at least 0.95 for the test dataset. The comparison AUC of mentioned tools and RF developed model showed that the performance of RF models is 11% to 30% higher than the 10 mentioned models. Moreover, the kappa coefficient indicated that there is as strong agreement between the 190 pairs of the test dataset. All of these indices showed that the developed model has a proper performance to predict the class of peptides.

The experimental studies on epitope of human showed that the epitopes contain hydrophobic, aliphatic or aromatic AA at positions 1, 4, 6, and 9 (43,44). The hydrophobic AA is the priority at position 1 and 9 (45–47). Six extracted decision rules in RF models for discriminate to class of peptide. Based on the results of this study, the developed RF model is highly efficient in the prediction of parasite MHC class II T cell epitopes.

## Conclusion

The random forest algorithm is a flexible, robustness and accurate statistical approach. This method is able to handle unbalanced datasets; many input features without variable deletion, estimates important scores for each feature without any required assumption and restriction in the traditional statistical methods. These advantages make it the most common method for classification of peptides. In the current study, an RF model was developed based on biochemical peptide properties to identify the class of peptides exist in a given protein. The performance measures of RF developed model improve in comparison with the common T-cell epitopes prediction tools. Accordingly, using the RF model facilitates selection of most likely immunogenic epitopes for further complementary experimental studies.

## Ethical considerations

Ethical issues (Including plagiarism, informed consent, misconduct, data fabrication and/or falsification, double publication and/or submission, redundancy, etc.) have been completely observed by the authors.

## References

[1] Kaufmann SH Juliana McElrath M (2014). Challenges and responses in human vaccine development. Curr Opin Immunol, 28 (1):18–26. 24561742 10.1016/j.coi.2014.01.009

[2] Flower DR (2014). Computer-Aided Vaccine Design. Hum Vaccines Immunother, 10 (1): 241–43. 10.4161/hv.26687 PMC4181008 24100661

[3] Kuleš J Horvatić A Guillemin N (2016). New approaches and omics tools for mining of vaccine candidates against vector-borne diseases. Mol Biosyst, 12 (9): 2680–94. 27384976 10.1039/c6mb00268d

[4] Soria-Guerra RE Nieto-Gomez R Govea-Alonso DO Rosales-Mendoza S (2015). An overview of bioinformatics tools for epitope prediction: Implications on vaccine development. J Biomed Inform, 53 (1): 405–14. 25464113 10.1016/j.jbi.2014.11.003

[5] Sanchez-Trincado JL Gomez-Perosanz M Reche PA (2017). Fundamentals and Methods for T- and B-Cell Epitope Prediction. J Immunol Res 2017:2680160. 29445754 10.1155/2017/2680160 PMC5763123

[6] Kar P Ruiz-Perez L Arooj M Mancera RL (2018). Current methods for the prediction of T-cell epitopes. Pept Sci, 110 (2) :e24046.

[7] Luo J Wu M Gopukumar D Zhao Y (2016). Big Data Application in Biomedical Research and Health Care: A Literature Review. Biomed Inform Insights, 8:1–10. 10.4137/BII.S31559 PMC4720168 26843812

[8] Chapter: Pang Shaoning Havukkala Ilkka Hu Yingjie Kasabov Nikola (2008). Bootstrapping Consistency Method for Optimal Gene Selection from Microarray Gene Expression Data for Classification Problems. In: Machine learning in bioinformatics. Eds, Zhang Rajapakse 1st ed, John Wiley & Sons Inc. Hoboken, NJ pp.: 89–110.

[9] Larrañaga P Calvo B Santana R (2006). Machine learning in bioinformatics. Brief Bioinform, 7 (1):86–112. 16761367 10.1093/bib/bbk007

[10] Chapter: Lata S Bhasin M Raghava GP (2007). Application of Machine Learning Techniques in Predicting MHC Binders. Methods Mol Biol, 409:201–15. 18450002 10.1007/978-1-60327-118-9_14

[11] Breiman L. Bagging predictors. (1996). Mach Learn, 24 (2):123–40.

[12] Chapter: Qi Yanjun (2012). Random Forests for Bioinformatics. In: Ensemble Machine Learning: Methods and Applications. Eds, Zhang Ma 1st ed, Springer-Verlag, New York pp.: 307–23.

[13] Chapter: Hastie T Tibshirani R Friedman J (2009). Boosting and Additive Trees. In: The Elements of Statistical Learning: Data Mining, Inference, and Prediction. Eds, Hastie Friedman 2nd ed, Springer-Verlag, New York pp.: 337–87.

[14] Chen X Wang M Zhang H (2011). The use of classification trees for bioinformatics. Wiley Interdiscip Rev Data Min Knowl Discov, 1 (1):55–63. 22523608 10.1002/widm.14 PMC3329156

[15] Breiman L (2001). Random Forests. Mach Learn, 45 (1):5–32.

[16] The UniProt Consortium (2008). The Universal Protein Resource (UniProt). Nucleic Acids Res, 36 (Database issue):D190–5. 18045787 10.1093/nar/gkm895 PMC2238893

[17] Apweiler R Bairoch A Wu CH (2004). Uni-Prot: the Universal Protein knowledgebase. Nucleic Acids Res, 32 (Database issue):D115–9. 14681372 10.1093/nar/gkh131 PMC308865

[18] Vita R Overton JA Greenbaum JA (2015). The immune epitope database (IEDB) 3.0. Nucleic Acids Res, 43(Database issue):D405–12. 25300482 10.1093/nar/gku938 PMC4384014

[19] Wang J Huda A Lunyak VV Jordan IK (2010). A Gibbs sampling strategy applied to the mapping of ambiguous short-sequence tags. Bioinformatics, 26 (20):2501–8. 20871106 10.1093/bioinformatics/btq460 PMC2951085

[20] Nielsen M Lundegaard C Worning P (2004). Improved prediction of MHC class I and class II epitopes using a novel Gibbs sampling approach. Bioinformatics, 20 (9):1388–1397. 14962912 10.1093/bioinformatics/bth100

[21] Ikai A (1980). Thermostability and aliphatic index of globular proteins. J Biochem, 88 (6):1895–8. 7462208

[22] Kyte J Doolittle RF (1982). A simple method for displaying the hydropathic character of a protein. J Mol Biol, 157 (1):105–32. 7108955 10.1016/0022-2836(82)90515-0

[23] Grantham R (1974). Amino Acid Difference Formula to Help Explain Protein Evolution. Science, 185 (4154):862–4. 4843792 10.1126/science.185.4154.862

[24] Kozlowski LP (2017). Proteome-pI: proteome isoelectric point database. Nucleic Acids Res, D1112–6. 27789699 10.1093/nar/gkw978 PMC5210655

[25] Zimmerman JM Eliezer N Simha R (1968). The characterization of amino acid sequences in proteins by statistical methods. J Theor Biol, 21 (2):170–201. 5700434 10.1016/0022-5193(68)90069-6

[26] Book: Barrett GC Elmore DT (1998). Physico-chemical properties of amino acids and peptides. In: Amino Acids and Peptides. 1st ed Cambridge University Press, Cambridge pp.: 32–46.

[27] Fawcett T (2006). An introduction to ROC analysis. Pattern Recognittion Letters, 27 (8):861–74.

[28] Mandrekar JN (2010). Receiver Operating Characteristic Curve in Diagnostic Test Assessment. J Thorac Oncol, 5 (9):1315–6. 20736804 10.1097/JTO.0b013e3181ec173d

[29] Chapter: Friedman CP Wyatt J (2006). Analyzing the Results of Demonstration Studies. In: Evaluation Methods in Biomedical Informatics. Eds, Kathryn Marion 2nd ed, Springer-Verlag, New York, pp: 224–47.

[30] Book: Altman DG (1990). Practical Statistics for Medical Research. 2nd ed Chapman and Hall/CRC, Boca Raton, Fla, p.:149–93.

[31] Book: Bland M (2000). An Introduction to Medical Statistics. 3rd ed Oxford University Press, New York, pp.: 47–66.

[32] Dimitrov I Garnev P Flower DR Doytchinova I (2010). EpiTOP—a proteochemometric tool for MHC class II binding prediction. Bioinformatics, 26 (16):2066–8. 20576624 10.1093/bioinformatics/btq324

[33] Guan P Doytchinova IA Zygouri C Flower DR (2003). MHCPred: a server for quantitative prediction of peptide–MHC binding. Nucleic Acids Res, 31 (13):3621–4. 12824380 10.1093/nar/gkg510 PMC168917

[34] Mustafa AS Shaban FA (2006). ProPred analysis and experimental evaluation of promiscuous T-cell epitopes of three major secreted antigens of Mycobacterium tuberculosis. Tuberculosis (Edinb), 86 (2):115–24. 16039905 10.1016/j.tube.2005.05.001

[35] Zhang L Chen Y Wong H-S (2012). TEPITOPEpan: Extending TEPITOPE for Peptide Binding Prediction Covering over 700 HLA-DR Molecules. PLOS ONE, 7 (2):e30483. 22383964 10.1371/journal.pone.0030483 PMC3285624

[36] Guan P Hattotuwagama CK Doytchinova IA Flower DR (2006). MHCPred 2.0: an updated quantitative T-cell epitope prediction server. Appl Bioinformatics, 5 (1):55–61. 16539539 10.2165/00822942-200605010-00008

[37] Liu W Meng X Xu Q Flower DR Li T (2006). Quantitative prediction of mouse class I MHC peptide binding affinity using support vector machine regression (SVR) models. BMC Bioinformatics, 7 (1):182. 16579851 10.1186/1471-2105-7-182 PMC1513606

[38] Dönnes P Kohlbacher O (2006). SVMHC: a server for prediction of MHC-binding peptides. Nucleic Acids Res, 34:W194–W197. 16844990 10.1093/nar/gkl284 PMC1538857

[39] Bhasin M Raghava GPS (2004). SVM based method for predicting HLA-DRB1*0401 binding peptides in an antigen sequence. Bioinformatics, 20 (3):421–3. 14960470 10.1093/bioinformatics/btg424

[40] Reche PA Glutting J-P Zhang H Reinherz EL (2004). Enhancement to the RANKPEP resource for the prediction of peptide binding to MHC molecules using profiles. Immunogenetics, 56 (6):405–19. 15349703 10.1007/s00251-004-0709-7

[41] Andreatta M Schafer-Nielsen C Lund O (2011). NNAlign: A Web-Based Prediction Method Allowing Non-Expert End-User Discovery of Sequence Motifs in Quantitative Peptide Data. PLOS ONE, 6 (11):e26781. 22073191 10.1371/journal.pone.0026781 PMC3206854

[42] Karosiene E Rasmussen M Blicher T (2013). NetMHCIIpan-3.0, a common pan-specific MHC class II prediction method including all three human MHC class II isotypes, HLADR, HLA-DP and HLA-DQ. Immunogenetics, 65 (10): 711–24. 23900783 10.1007/s00251-013-0720-y PMC3809066

[43] Brown JH Jardetzky TS Gorga JC (1993). Three-dimensional structure of the human class II histocompatibility antigen HLA-DR1. Nature, 364 (1):33–39. 8316295 10.1038/364033a0

[44] Painter CA Stern LJ (2012). Conformational variation in structures of classical and non-classical MHCII proteins and functional implications. Immunol Rev, 250 (1):144–57. 23046127 10.1111/imr.12003 PMC3471379

[45] Mommen GPM Marino F Meiring HD (2016). Sampling From the Proteome to the Human Leukocyte Antigen-DR (HLA-DR) Ligandome Proceeds Via High Specificity. Mol Cell Proteomics, 15 (4):1412–23. 26764012 10.1074/mcp.M115.055780 PMC4824864

[46] Sidney J Steen A Moore C (2010). Five HLA-DP Molecules Frequently Expressed in the Worldwide Human Population Share a Common HLA Supertypic Binding Specificity. J Immunol, 184 (5):2492–503. 20139279 10.4049/jimmunol.0903655 PMC2935290

[47] Sidney J Steen A Moore C (2010). Divergent Motifs but Overlapping Binding Repertoires of Six HLA-DQ Molecules Frequently Expressed in the Worldwide Human Population. J Immunol, 185 (7):4189–98. 20810981 10.4049/jimmunol.1001006 PMC3307390

